# Graphene–Liquid Crystal Synergy: Advancing Sensor Technologies across Multiple Domains

**DOI:** 10.3390/ma17174431

**Published:** 2024-09-09

**Authors:** Mohammad A. Adeshina, Abdulazeez M. Ogunleye, Hakseon Lee, Bharathkumar Mareddi, Hyunmin Kim, Jonghoo Park

**Affiliations:** 1 School of Electronic and Electrical Engineering, Kyungpook National University, Daegu 41566, Republic of Korea; mohammadadeshina1@gmail.com (M.A.A.);; 2 Division of Biotechnology, Daegu Gyeongbuk Institute of Science and Technology (DGIST), Daegu 42988, Republic of Korea; 3 Electronics and Communication Engineering, Christ University, Bangalore 560074, India; 1992mbkr@gmail.com

**Keywords:** liquid crystal, graphene, sensors

## Abstract

This review explores the integration of graphene and liquid crystals to advance sensor technologies across multiple domains, with a focus on recent developments in thermal and infrared sensing, flexible actuators, chemical and biological detection, and environmental monitoring systems. The synergy between graphene’s exceptional electrical, optical, and thermal properties and the dynamic behavior of liquid crystals leads to sensors with significantly enhanced sensitivity, selectivity, and versatility. Notable contributions of this review include highlighting key advancements such as graphene-doped liquid crystal IR detectors, shape-memory polymers for flexible actuators, and composite hydrogels for environmental pollutant detection. Additionally, this review addresses ongoing challenges in scalability and integration, providing insights into current research efforts aimed at overcoming these obstacles. The potential for multi-modal sensing, self-powered devices, and AI integration is discussed, suggesting a transformative impact of these composite sensors on various sectors, including health, environmental monitoring, and technology. This review demonstrates how the fusion of graphene and liquid crystals is pushing the boundaries of sensor technology, offering more sensitive, adaptable, and innovative solutions to global challenges.

## 1. Introduction

The convergence of graphene and liquid crystals has emerged as a promising frontier in advanced sensor technologies, offering unprecedented opportunities for innovation across multiple domains. This powerful combination leverages the unique properties of both materials to create novel sensing platforms with enhanced performance and versatility. Graphene, a two-dimensional carbon allotrope, has garnered significant attention since its isolation in 2004 due to its exceptional electrical, mechanical, and optical properties [1,2]. Its high carrier mobility, optical transparency, and mechanical strength make it an ideal candidate for various sensing applications [3,4,5,6]. The ability to engineer graphene at the nanoscale with specific properties and architectures has opened new avenues for creating customized graphene-based materials tailored for diverse applications.

**Figure 1:**
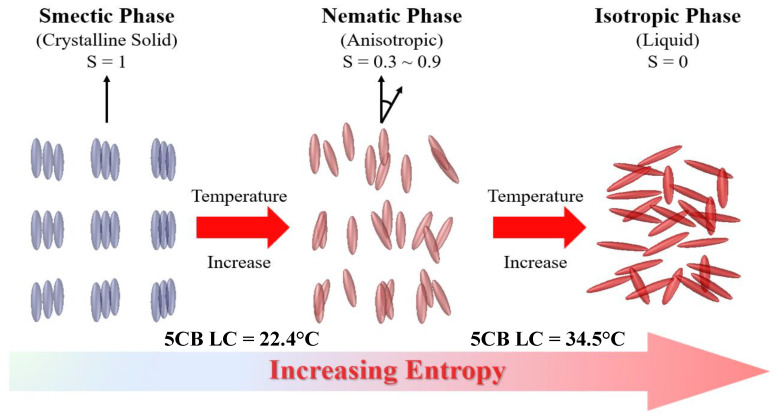
Three phases of a thermotropic liquid crystal.

Liquid crystals represent a unique state of matter that combines properties of both liquids and solids. They exhibit an ordered molecular arrangement characteristic of solids while maintaining the ability to flow like liquids [7,8,9]. These materials are composed of anisotropic molecules, typically elongated and rod-like or disk-shaped, which possess different optical and physical properties along different directions. This anisotropy stems from the molecules’ preferred alignment direction, known as the director. A key feature of liquid crystals is their high responsiveness to external stimuli such as electric or magnetic fields, heat, or light [10,11,12]. These stimuli can cause the director to rotate or reorient, leading to changes in the material’s optical, electrical, or mechanical properties. As shown in Figure 1, thermotropic liquid crystals exhibit three distinct phases, isotropic, nematic, and smectic, which are largely dependent on temperature.

**Figure 2:**
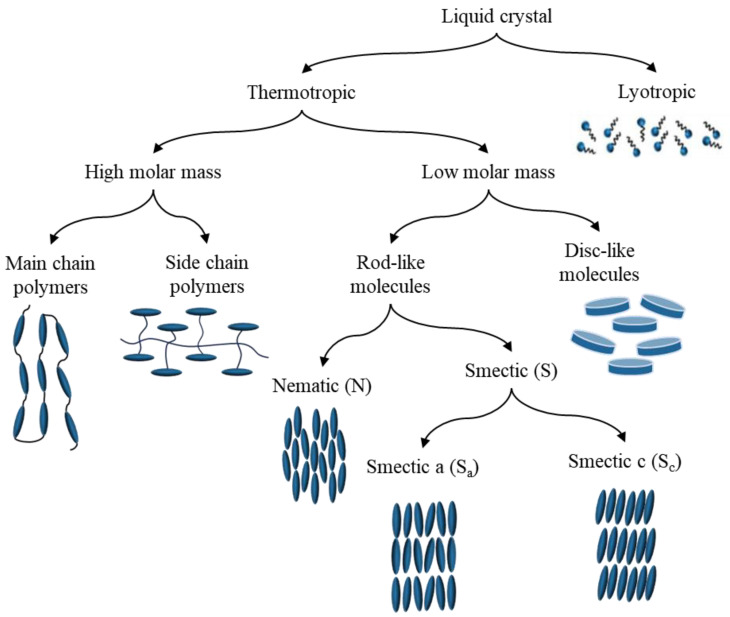
General classification of most popular liquid crystal phases.

Figure 2 illustrates the broad classification of the most popular liquid crystal phases. Liquid crystals are primarily categorized into two types: thermotropic and lyotropic. Thermotropic liquid crystals undergo phase transitions between solid, liquid crystal, and liquid states in response to temperature changes. The temperature at which this phase transition occurs, known as the clearing temperature, is influenced by the chemical structure of the liquid crystal molecules. This temperature can be adjusted by modifying the length and chemical composition of the alkyl chains or aromatic rings within the molecule. In contrast, lyotropic liquid crystals form when amphiphilic molecules, such as lipids and surfactants, are dissolved in a solvent, typically water under specific concentration, temperature, and pressure conditions. These amphiphilic molecules self-assemble into ordered aggregates with distinct shapes and sizes, which then organize themselves into ordered phases. The formation of these ordered phases is primarily dependent on the concentration of the amphiphilic molecules and the nature of the solvent [13,14,15,16].

Liquid crystals are further categorized based on their molecular structure and mesophase organization. High molar mass liquid crystals, also known as polymeric liquid crystals, are composed of long-chain molecules exhibiting a high degree of order in their arrangement. These are subdivided into main-chain liquid crystals (rigid, rod-like molecules connected by flexible spacer units) and side-chain liquid crystals (flexible, chain-like molecules attached to a rigid, planar core structure). Main-chain liquid crystals demonstrate a higher degree of order due to their greater molecular rigidity, while side-chain liquid crystals allow for easier packing but may introduce some disorder due to the flexibility of their side chains. Low molar mass liquid crystals, or small molecule liquid crystals, consist of smaller molecules with a more disordered arrangement. These are further divided into discotic (disk-shaped) and rod-shaped liquid crystals. Discotic liquid crystals, composed of disk-shaped molecules, form a 2D hexagonal or columnar structure and possess unique electronic and optical properties, making them suitable for applications in electronic sensors and other devices [17,18,19].

Rod-shaped liquid crystals include nematic and smectic types. Nematic liquid crystals have a one-dimensional (1D) long-range order but no short-range order, with molecules aligning along the director while retaining rotational freedom. This results in a fluid-like material with anisotropic properties along the director’s direction. Smectic liquid crystals form a two-dimensional (2D) layered structure with a long-range order within each layer but no order between layers. They are further classified into Smectic A (molecules oriented perpendicular to the layer) and Smectic C (molecules tilted with respect to the layer). The unique molecular orientation, responsiveness to external stimuli, and self-assembly capabilities of liquid crystals provide a dynamic platform for creating ordered structures at the nanoscale [11,14,15,20,21,22]. This comprehensive classification system provides a framework for understanding the diverse structures and properties of liquid crystals, which is crucial for their various applications in sensing technologies and beyond.

**Figure 3:**
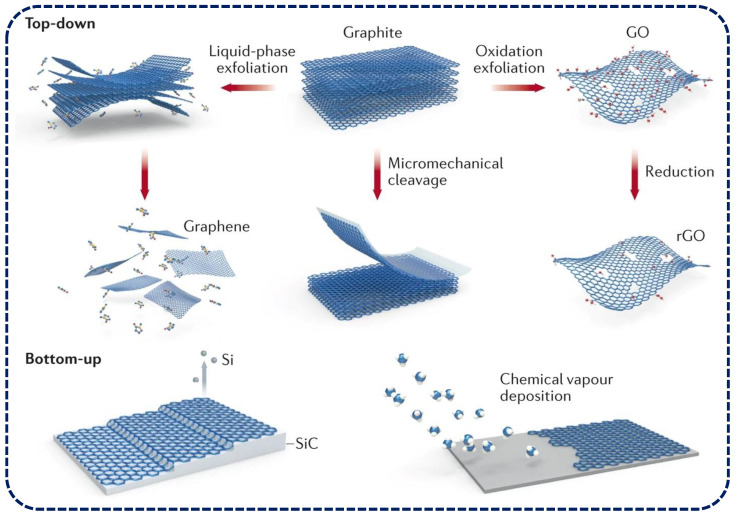
General strategies for the synthesis of graphene and its derivatives. Ref. [25] reproduced with permission from Springer Nature.

The integration of graphene with liquid crystals combines the sensitivity and conductivity of graphene with the self-organizing and stimuli-responsive nature of liquid crystals. Graphene’s key properties, including its high electrical conductivity, mechanical strength, and large surface area, make it highly suitable for sensor applications. Graphene’s honeycomb lattice structure serves as an effective tool for aligning liquid crystal (LC) molecules, primarily due to π–π interactions between graphene’s sp^2^ carbon atoms and the aromatic rings of the nematic LC [11]. The combination of graphene with liquid crystals enhances sensor sensitivity and selectivity, though challenges such as achieving uniform dispersion and maintaining stability in the liquid crystal matrix remain. This pairing has led to the development of advanced sensors capable of detecting a wide range of physical, chemical, and biological stimuli with high sensitivity and specificity. As shown in Figure 3, recent advancements in synthesis techniques have further expanded the potential of graphene–liquid crystal composites. Methods such as chemical vapor deposition (CVD), liquid-phase exfoliation, and reduction of graphene oxide have enabled the production of high-quality graphene materials suitable for integration with liquid crystals [3,23,24,25]. These techniques allow for precise control over the graphene structure, from single-layer sheets to few-layer assemblies, each with distinct properties that can be tailored for specific sensing applications.

This review paper aims to explore the cutting-edge developments in graphene–liquid crystal composite sensors across multiple domains. We will examine their applications in thermal and infrared sensing, flexible and wearable actuators, chemical and biological detection, and environmental monitoring systems. By analyzing the combined effects of graphene and liquid crystals, we seek to highlight the transformative potential of these composite materials in advancing sensor technologies. Furthermore, we will discuss the challenges and limitations currently faced in this field and provide insights into future research directions. As we delve into the various applications and underlying mechanisms, we aim to provide a comprehensive overview of how graphene–liquid crystal integration is pushing the boundaries of sensor technology and paving the way for next-generation sensing devices.

## 2. Graphene–Liquid Crystal Composites in Sensor Applications

The integration of graphene and liquid crystals creates innovative sensing platforms that leverage the unique properties of both materials. Graphene has exceptional electrical, mechanical, and optical characteristics, while liquid crystals offer dynamic responsiveness and self-organizing capabilities. This synergy results in highly sensitive and versatile sensors applicable across various domains.

### 2.1. Thermal and Infrared Sensing

**Figure 4:**
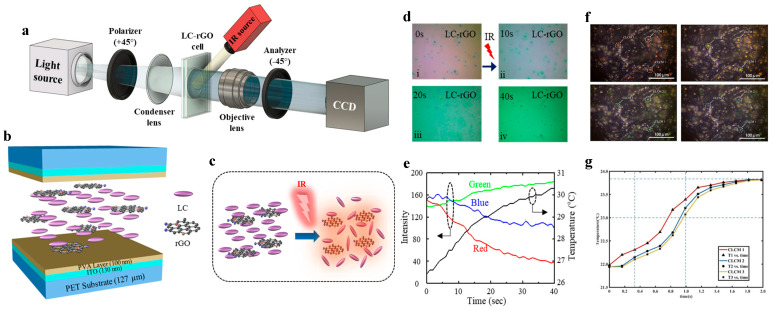
Graphene–liquid crystal composites for IR detection and temperature sensing. (**a**) Schematic of IR detector setup: LC-rGO cell, polarized optical microscope (POM), and IR source. (**b**) LC-rGO cell structure. (**c**) Diagram illustrating photothermal effect on LC-rGO orientational disorder under IR irradiation. (**d**) Time-lapse POM images of LC-rGO cells at 0–40 s (**i**–**iv**) during IR exposure. (**e**) RGB time-evolution and temperature rise analysis of LC-rGO cell [26]. (**f**) Microscopic temperature visualization in porous graphene using cholesteric liquid crystal microcapsules (CLCMs), showing heat transfer from lower right corner. (**g**) Comparative thermal response data for various LC configurations [27] reproduced with permission.

Graphene’s exceptional thermal and electrical properties make it an ideal candidate for enhancing the sensitivity and response time of IR detectors. In this review, we consider scenarios where graphene does not chemically interact with liquid crystal molecules, focusing instead on phase transitions driven by physical phenomena such as freezing point depression or photothermal effects. In the context of a liquid phase IR detector, reduced graphene oxide (rGO)-doped liquid crystals (LCs) are utilized to exploit the photothermal effect [26]. The setup involves an IR source directed at an LC-rGO cell, which is observed through a polarized optical microscope (POM). The schematic diagram in Figure 4a illustrates the components of this setup, highlighting the interaction between the IR radiation and the LC-rGO composite. The structural diagram in Figure 4b shows the layered arrangement within the LC-rGO cell, where the graphene flakes are dispersed within the liquid crystal matrix. Upon IR irradiation, these graphene flakes absorb the IR energy and convert it into heat. This heat induces a photothermal effect, causing a change in the orientation and disorder of the liquid crystals, as shown in Figure 4c. The time-lapse images shown in Figure 4d capture the dynamic changes within the LC-rGO cell, showcasing the gradual thermal response from 0 to 40 s. The RGB time-evolution graph shown in Figure 4e quantitatively depicts the colorimetric response of the liquid crystals, correlating it with the temperature rise in the cell. This analysis underscores the potential of LC-rGO composites in providing real-time and precise IR detection capabilities. In another innovative application, cholesteric liquid crystal microcapsules (CLCMs) are used to visually measure the microscopic temperature of porous graphene [27]. The ability of CLCMs to exhibit distinct color changes in response to temperature variations makes them suitable for high-resolution thermal mapping. The visualization process involves dispersing CLCMs over a porous graphene substrate. As shown in Figure 4f, the heat transfer from a localized source (typically the lower right corner) causes a gradual color shift in the CLCMs, indicating temperature changes. This method provides a non-invasive and highly visual approach to monitor microscopic temperature variations across the graphene surface. Comparative thermal response data in Figure 4g further demonstrate the effectiveness of different liquid crystal configurations in responding to thermal stimuli. The data indicate that the integration of graphene enhances the thermal sensitivity and response time of the liquid crystals, making them more efficient for temperature-sensing applications. Both these innovative approaches showcase the immense potential of graphene–liquid crystal composites in thermal and IR sensing. The rGO-doped liquid crystal detector offers high sensitivity and rapid response for precise IR detection, while the cholesteric liquid crystal microcapsules provide a unique solution for visual temperature mapping. Additionally, a study on a temperature sensor design based on a photonic crystal with a defect layer of graphene monolayers deposited on nematic liquid crystal demonstrates high sensitivity and quality factor, offering a sensitivity of 4 nm °C^−1^ and a quality factor of up to 11,000 [28]. These studies have provided numerical estimations of the heat absorption capacity of graphene when introduced into liquid crystal matrices. The insights enable the strategic design of devices where graphene concentration dictates phase behavior, thus broadening the potential for innovation in sensor technology development. These advancements are paving the way for new applications across various fields, including thermal imaging, non-destructive testing, and biomedical diagnostics.

### 2.2. Flexible and Wearable Actuators

**Figure 5:**
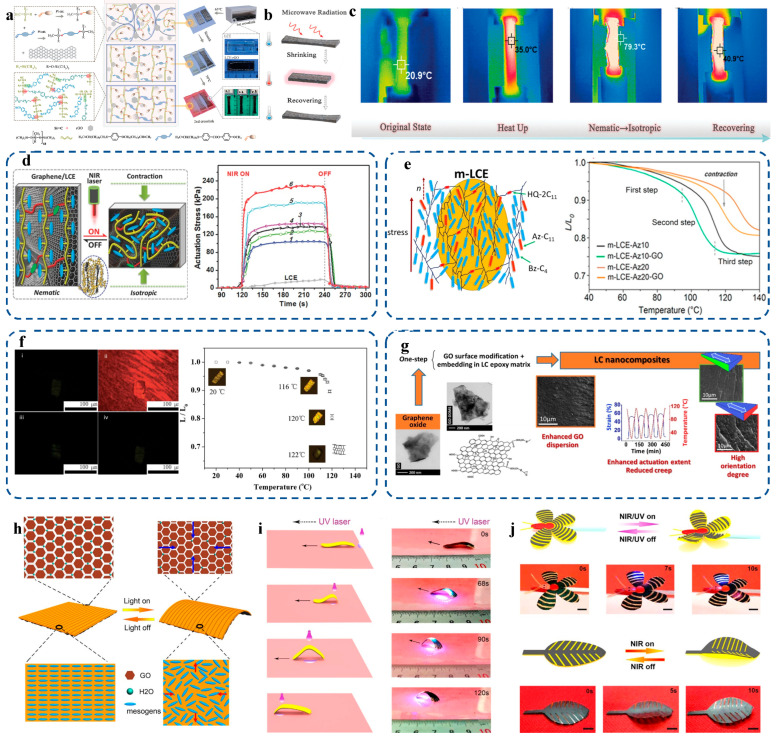
(**a**) Preparation and characterization of LCE-rGO composites, showing precursor reactants, cross-linked matrix network formation, monodomain state formation in nematic phase polymer network, and optical images of partially and fully cross-linked LCE-rGO. (**b**) Reversible memory deformation of LCE-rGO by microwave irradiation. (**c**) Thermal infrared images of LC-rGO during whole microwave drive. Ref. [29] reproduced with permission. (**d**) Schematic of reversible photomechanical actuation and actuation stress response in graphene/LCE nanocomposites under NIR light. Ref. [30] reproduced with permission. (**e**) Thermo-elastic behavior of modified liquid crystal elastomers. Ref. [31] reproduced with permission. (**f**) LCE micropillar in 118 °C silicon oil with laser on/off and length change in LCE pillars with temperature, with POM insets at different temperatures. Ref. [32] reproduced with permission. (**g**) Shape-memory characteristics of liquid-crystalline elastomer/graphene oxide nanocomposites. Ref. [33] reproduced with permission. (**h**) Schematic of light-driven deformation in bilayer films, with isotropic shrinkage in GO layer and anisotropic contraction in ALCN layer. (**i**) Crawling robot with ALCN outer layer and GO inner layer, responding to UV light. Soft robot and foot-shaped actuator move along UV laser direction, indicated by black arrows. (**j**) Biomimetic behaviors of GO-ALCN microrobots with concentric and leaf-like GO layers under UV/NIR light, showing real-time light-controlled deformations. Ref. [34] reproduced with permission. Copyright 2020 American Chemical Society.

The combination of graphene with liquid crystals has revolutionized the field of flexible and wearable actuators, providing enhanced performance through their tunable properties and responsive behaviors under various stimuli. This synergy is vividly demonstrated across multiple applications, as illustrated by recent studies. Graphene-implanted shape-memory polymers exhibit remarkable efficiency in actuation under microwave stimuli. Figure 5a–c detail the preparation and characterization of liquid crystal elastomer (LCE) composites with reduced graphene oxide (rGO), highlighting the formation of a cross-linked matrix network and the development of a monodomain state in the nematic phase. These composites demonstrate reversible memory deformation and significant actuation stress under NIR irradiation, showcasing their potential in flexible electronics and adaptive materials [29]. Further advancements are seen in graphene-enabled photomechanical actuation in LCE nanocomposites. Figure 5d illustrates the reversible actuation and stress response under NIR light, underscoring the enhanced elastic and responsive properties conferred by graphene. This makes them suitable for precise control applications in wearable devices and soft robotics [30].

The incorporation of gold and graphene oxide nanoparticles into monodomain liquid crystal elastomers enhances their thermo- and photo-actuation capabilities, as shown in Figure 5e. These modified elastomers exhibit significant actuation upon exposure to temperature changes or light, making them ideal for flexible actuators that adapt to environmental conditions [31]. Photo-responsive LCEs with reduced chemically modified graphene oxide also display impressive shape-memory characteristics. Figure 5f shows significant length changes in LCE micropillars with temperature variations and POM images of structural changes [32]. These materials offer reliable and repeatable movements, which are crucial for flexible and wearable actuators. The shape-memory characteristics of liquid-crystalline elastomer/graphene oxide nanocomposites demonstrate enhanced actuation and reduced creep, as shown in Figure 5g [33]. These properties make them highly effective for flexible actuators requiring reliable and repeatable movements, underscoring their potential for advanced wearable technologies. Lastly, NIR-UV responsive actuators utilizing GO/microchannel-induced liquid crystal bilayer structures present advanced applications in biomimetic devices. Figure 5h–j demonstrate light-driven deformation, isotropic and anisotropic shrinkage, and responsive movement in actuators, mimicking natural behaviors. These actuators promise significant advancements in developing life-like, adaptive wearable devices [34]. The synergy between graphene and liquid crystals presents a versatile platform for flexible and wearable actuators, offering tunable actuation, shape memory, and responsive behaviors to light and temperature. These properties enable innovative applications in soft robotics, adaptive materials, and wearable technology, marking a significant advancement in sensor technologies.

### 2.3. Chemical and Biological Detection

**Figure 6:**
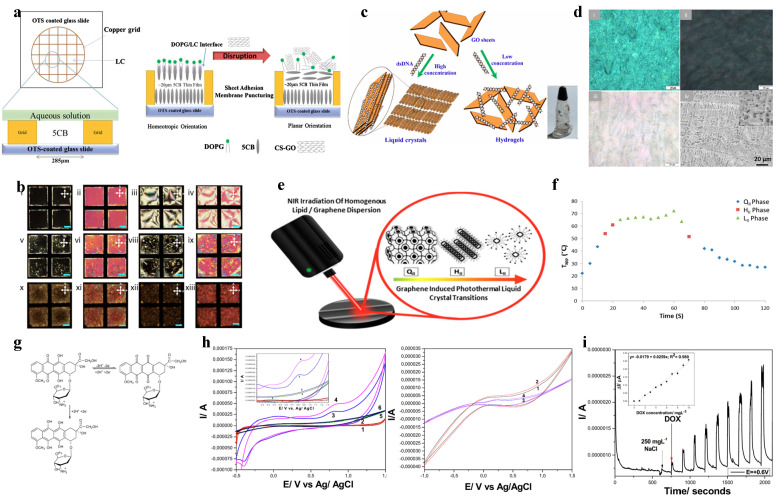
(**a**) Schematic of the setup for inducing lipid liquid-crystal phase change through near-infrared irradiation of graphene particles. (**b**) Polarized optical microscopy images showing the orientation disruption of liquid crystals due to graphene oxide interaction. Ref. [35] reproduced with permission. (**c**) Diagram illustrating the synthesis of graphene oxide/double-stranded DNA composite liquid crystals and hydrogels. (**d**) Optical and SEM images depicting the structural characteristics of these hydrogels. Ref. [36] reproduced with permission. (**e**) Setup for graphene-induced photothermal liquid crystal transitions and (**f**) the corresponding temperature–time response graph showing different phases during IR exposure. Ref. [37] reproduced with permission Copyright 2015 American Chemical Society. (**g**) Chemical structures of doxorubicin and its electrochemical derivatives. (**h**) Time-dependent current response for detecting doxorubicin, highlighting the sensor’s sensitivity. (**i**) Cyclic voltammetry graphs demonstrating the electrochemical detection capabilities of doxorubicin using graphene quantum dots and Cu(I) liquid crystals. Ref. [38] reproduced with permission under Creative Commons license.

Graphene’s exceptional photothermal properties are harnessed in chemical and biological sensors, as illustrated in Figure 6a. The setup involves lipid liquid crystals undergoing phase changes when exposed to near-infrared (NIR) irradiation of graphene particles. This irradiation generates heat due to graphene’s photothermal effect, altering the liquid crystal’s orientation and phase. These changes are easily detectable through optical methods, enabling the development of highly sensitive thermal sensors. Such sensors can be used for real-time temperature monitoring in various chemical and biological environments. The interaction between graphene oxide (GO) and liquid crystals (LCs) plays a crucial role in the development of biological sensors. GO’s ability to disrupt the orientation of LCs, as shown in Figure 6b, is critical for detecting biological interactions, such as those with phospholipid membranes. This disruption, observable through polarized optical microscopy, provides valuable insights into the presence and activity of various biological substances, including antibacterial agents. This disruption indicates the effect of GO on phospholipid membranes, which is crucial for developing sensors that monitor antibacterial activity and other biological interactions. These interactions lead to structural changes in the liquid crystals, providing valuable data on the presence and activity of various substances [35].

The synthesis process of graphene oxide (GO) and double-stranded DNA (dsDNA) composites results in liquid crystals and hydrogels, as shown in Figure 6c,d. These composites exhibit unique structural characteristics, enhancing their use in various sensing applications. Hydrogels, known for their absorbent and responsive nature, benefit from the incorporation of GO, which improves their mechanical and electrical properties. This enhancement makes them ideal for environmental monitoring and biosensing applications, where detecting and responding to chemical and biological stimuli is crucial [36]. The use of cholesteric liquid crystal microcapsules (CLCMs) for visual temperature measurement of porous graphene has been demonstrated. The setup in Figure 6e shows graphene-induced photothermal liquid crystal transitions, where the liquid crystals change phase upon IR exposure. The temperature–time response graph (Figure 6f) depicts different phases during IR exposure, illustrating the liquid crystal’s sensitivity to temperature changes. This method provides a highly visual and non-invasive approach to monitoring microscopic temperature variations across the graphene surface, which is essential for applications requiring precise thermal monitoring [37].

Furthermore, the advanced electrochemical detection capabilities of graphene quantum dots (GQDs) integrated with Cu(I) liquid crystals are illustrated in Figure 6g–i. The chemical structure of doxorubicin (Figure 6g), the time-dependent current response (Figure 6h), and the cyclic voltammetry graphs (Figure 6i) highlight the sensor’s sensitivity and efficiency in detecting doxorubicin in aqueous solutions. The high surface area and excellent electrical properties of GQDs improve the sensor’s electrochemical response, while their quantum confinement effects enhance selectivity, making this composite material ideal for precise drug monitoring in medical applications [38]. Integrating graphene and liquid crystals in sensor technology offers significant advancements in chemical and biological sensing. The unique properties of graphene, combined with the responsive nature of liquid crystals, create highly sensitive and versatile platforms for detecting various substances and monitoring biological activities. These innovations hold great potential for applications in medical diagnostics, environmental monitoring, and beyond, paving the way for more efficient and precise sensing technologies.

### 2.4. Environmental Monitoring Systems

**Figure 7:**
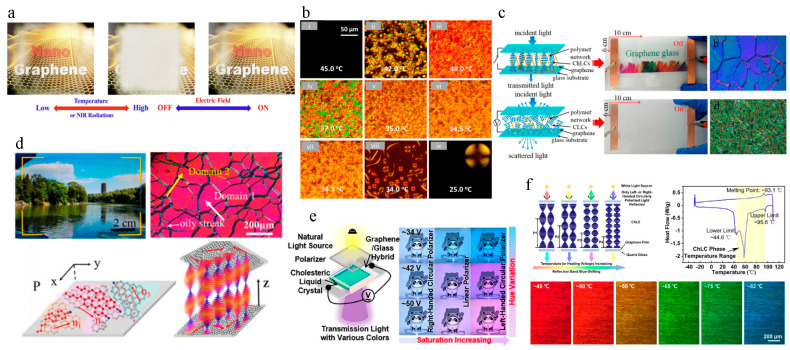
(**a**) Schematic of the graphene-based system showing the effect of temperature and electric fields for smart window application. (**b**) Phase transitions of a chiral liquid crystal mixture observed with POM, from isotropic to N* and SmA* phases ranging from 25.0 °C to 45.0 °C. Ref. [39] reproduced with permission. (**c**) Diagram of light transmission and scattering in graphene and cholesteric liquid crystal (ChLC) composites, showing adaptive transparency: planar texture when off and focal conic texture when on, indicating potential for environmental monitoring. (**d**) Optical image demonstrating environmental clarity changes through a graphene-based filter system and structural and domain analysis of graphene/ChLC composites under polarized light. Ref. [40] reproduced with permission Copyright 2018 American Chemical Society. (**e**) Device showing hue and saturation changes in transmitted light with varying heating voltages and polarizations, applicable as visual indicators for environmental monitoring. (**f**) Diagram and differential scanning calorimetry (DSC) graph showing temperature range and phase transitions in ChLC/graphene systems. Sequential images depicting color change in the ChLC/graphene system at various temperatures, ranging from −45 °C to −82 °C, indicating different environmental conditions. Ref. [41] reproduced with permission Copyright 2023 American Chemical Society.

The amalgamation of graphene and liquid crystals offers groundbreaking advancements in environmental monitoring systems, as illustrated by various innovative applications. One notable application is the development of smart windows enabled by mesogen-functionalized graphene, which demonstrates a dynamic response to external stimuli. As illustrated in Figure 7a,b, these composites enable smart windows to transition between transparent and opaque states in response to external stimuli, such as NIR irradiation and electric fields. This capability allows for effective regulation of indoor lighting and temperature, thereby enhancing energy efficiency. The phase transitions observed in the chiral liquid crystal mixture, transitioning from isotropic to N* and SmA* phases, highlight the material’s thermal sensitivity, making it ideal for real-time environmental monitoring [39]. Further advancements are shown in Figure 7c,d, where graphene glass induces multidomain orientations in cholesteric liquid crystal (ChLC) devices. This fusion results in adaptive transparency, offering clear visual data under varying light conditions, which is crucial for environmental monitoring applications. The ability to switch between planar and focal conic textures enhances optical clarity and control, which is essential for high-performance environmental sensors [40].

The electro-driven thermochromic properties of graphene/ChLC composites, depicted in Figure 7e,f, enable dynamic light modulation and color tuning. These devices adjust the hue and saturation of transmitted light based on heating voltages and polarizations, providing real-time visual indicators of environmental changes. The temperature-dependent structural color variations in ChLCs, shifting from red to blue with rising temperature, demonstrate the potential for precise thermal monitoring, which is crucial for responsive environmental sensing [41]. Additionally, the alignment properties of nematic liquid crystal 5CB using graphene oxide provide a reference for creating highly sensitive environmental monitoring sensors. These sensors can detect subtle environmental changes through phase transitions, enhancing the overall effectiveness of graphene–liquid crystal composites in diverse monitoring applications [42]. The synergy between graphene and liquid crystals offers significant improvements in environmental monitoring technologies. The adaptive transparency, precise thermal sensitivity, and dynamic visual indicators of these systems underscore their potential to revolutionize sensor performance and expand applications in environmental monitoring, making them indispensable tools for advancing sensor technologies across multiple domains.

## 3. Challenges and Future Perspectives

The integration of graphene and liquid crystals in sensor technologies holds significant promise, but several challenges must be addressed for widespread adoption. One of the primary hurdles is scalability and cost. Current graphene production methods, such as chemical vapor deposition (CVD), are expensive and not yet feasible for mass production. Therefore, developing more economical synthesis techniques and optimizing merging processes are crucial steps toward commercial viability. Another challenge lies in the stability and longevity of these composites under various environmental conditions. To enhance durability, research is needed on protective coatings and encapsulation techniques. Additionally, improving selectivity in complex, real-world environments, where multiple interfering analytes are present, is an ongoing area of focus. Strategies such as surface functionalization or the incorporation of selective receptors are being explored to address this issue. Standardization of fabrication processes and characterization methods is also essential to ensure the reproducibility and comparability of results across different studies. Furthermore, integrating graphene–liquid crystal composites seamlessly with current electronic systems and data processing platforms remains a significant challenge but also an opportunity for innovation. Also, comparison with other state-of-the-art sensor technologies reveals that while traditional sensors such as silicon-based photodetectors and polymer-based flexible actuators offer robust performance, graphene–liquid crystal composites provide superior sensitivity, enhanced physical properties, and flexibility. Specifically, these composites outperform existing technologies in scenarios requiring high responsiveness to external stimuli, enhanced selectivity, and adaptability to various environmental conditions. These features make them suitable for a wide range of applications, including thermal and infrared sensing, flexible and wearable electronics, environmental monitoring, and telecommunications.

Looking ahead, the unique properties of graphene and liquid crystals offer exciting possibilities. Multi-modal sensing capabilities could lead to versatile platforms detecting various stimuli simultaneously. The flexibility and transparency of these composites make them ideal for wearable sensors and smart textiles. Exploring energy harvesting could result in self-powered sensors, while integrating artificial intelligence could enhance data interpretation and predictive capabilities. Biomedical applications and environmental monitoring could be revolutionized with further research into biocompatibility and functionalization. As research progresses, we anticipate increasingly sophisticated graphene–liquid crystal sensor technologies. Overcoming current challenges will pave the way for transformative applications across multiple domains. Ongoing advancements in materials science, nanotechnology, and data processing will contribute to realizing the full potential of this integration, promising more sensitive, responsive, and adaptable sensing solutions for the future.

## 4. Conclusions

The integration of graphene and liquid crystals has significantly advanced sensor technologies across multiple domains, including thermal and infrared sensing, flexible actuators, chemical and biological detection, and environmental monitoring. This combination leverages graphene’s unique properties and liquid crystals’ stimuli-responsive nature to create highly sensitive and versatile sensing platforms. While challenges in scalability and integration persist, ongoing research addresses these issues. The future of graphene–liquid crystal composite sensors is promising, with the potential for multi-modal sensing, self-powered devices, and AI integration. This synergy represents a paradigm shift in sensor technology, offering more sensitive and adaptable solutions for addressing global challenges in health, environment, and technology.
